# Why a New Journal?

**DOI:** 10.1155/S1064744993000018

**Published:** 1993

**Authors:** Sebastian Faro


Infectious Diseases in Obstetrics and Gynecology 1:1 (1993)

© 1993 Wiley-Liss, Inc.

### Why a New Journal?

Is there need for a journal of infectious diseases in obstetrics and gynecology?

It is true that the number of articles related to infectious diseases in obstetrics and gynecology published in existing journals has increased significantly. Many of these articles contain important information on antibiotics or the microbiology and pathophysiology as well as the epidemiology of infections that the practitioner of obstetrics and gynecology needs for the prevention of such diseases or diagnosis and treatment of patients affected by them. The primary care practitioner delivering health care to the female patient is also in need of this information. These articles are dispersed among various journals, however, and many of the journals are unrelated to the specialty of obstetrics and gynecology, making them not readily accessible to the obstetrician-gynecologist or the primary care physician.

The conception of this journal is a result of the need for a single reference source on the most current information on infectious diseases associated with the obstetric and gynecologic patient. To fulfill this need, *Infectious Diseases in Obstetrics and Gynecology* will publish material encompassing not only infectious diseases affecting the female reproductive organs, but every aspect of infectious diseases associated with the obstetric or gynecologic patient.

Each issue will contain 8 to 12 articles divided among basic science, clinical research, and clinical practice as they relate to infectious diseases in the female patient. Included in each issue will be a review article on an infectious disease subject related to obstetrics and gynecology. This review article will contain a synopsis of background information enabling the reader to develop a foundation of knowledge on the subject being discussed, information on current developments in the area, and management recommendations. Also included will be an obstetrical and a gynecological case presentation to serve as a stimulus for discussion on the management of problems commonly associated with our specialty, raising questions and issues for further discussion. The case presentations should also offer the reader the most current diagnostic tools and management regimens. Finally, each issue will contain an editorial dealing with a pertinent topic. The intent of this editorial is to stimulate questions from the readers and answers from various authorities that will be published in subsequent issues.

In summary, the purpose of this journal is to offer a forum for obtaining and disseminating information to clinicians and other health care providers, including researchers involved in the study or treatment of infectious diseases in the female patient as well as residents in training.

*Sebastian Faro, M.D., Ph.D.*
*Editor-in-Chief*

